# Supplementary material for: The ABCISIC ACID INSENSITIVE (ABI) 4 Transcription Factor Is Stabilized by Stress, ABA and Phosphorylation
Source: Plants (Basel). 2022 Aug 22;11(16):2179. doi: 10.3390/plants11162179 (PMC9414092; doi:10.3390/plants11162179)
Supplement: Supplementary file 1 [file plants-11-02179-s001.zip › Tzofia ABI4 Figure S1.pdf]

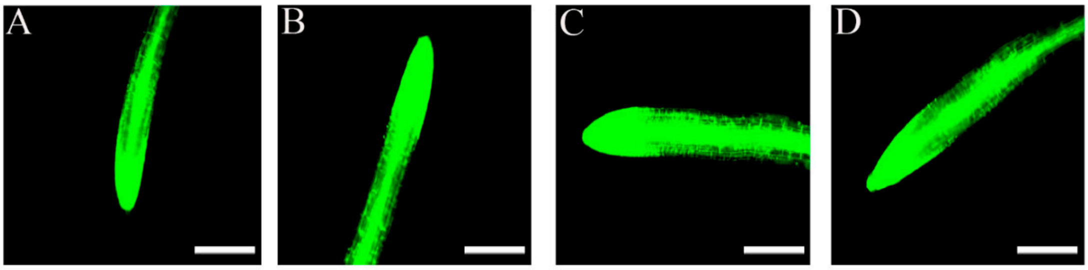

Figure S1. eGFP expression in roots of salt-treated *35s::eGFP* plants. Ten days old transgenic plants overexpressing *35S::eGFP* incubated for the indicated times with 0.5 x MS, 0.5% sucrose, without NaCl (A) or with 0.3 M NaCl, for 2.5 h (B), 4 h (C), 6 h (D). Roots were examined by fluorescence microscopy. Scale bar = 100 µm.
