# Supplementary material for: The ABCISIC ACID INSENSITIVE (ABI) 4 Transcription Factor Is Stabilized by Stress, ABA and Phosphorylation
Source: Plants (Basel). 2022 Aug 22;11(16):2179. doi: 10.3390/plants11162179 (PMC9414092; doi:10.3390/plants11162179)
Supplement: Supplementary file 1 [file plants-11-02179-s001.zip › Tzofia ABI4 Table S1.pdf]

Supplemental Table S1. Primers used in this study

No	Primer set	Forward primer	Reverse primer	Used for
1	pCAMBIA 1302	CTAATTCCTAAAACCAAAATCCAGTGACAATT AAACTATCAGTGTTTGACAGGATA	CATGTTGACCGGTTAGGGATAACAGTGCCTAATG AGTGAGCTAACTCAC	Construction of the pGA-eGFP plasmid
2	pSAT4	GTGAGTTAGCTCACTCATTAGGCACTGTTATC CCTAACCGGTCAACATG	TATCCTGTCAAACACTGATAGTTTAATTGTCACTG GATTTTGGTTTTAGGAATTAG	Construction of the pGA-eGFP plasmid
3	ABI4 SalI	GTCGACCTCGAGATGGACCCTTTAGCTTCCC	GTCGACTGCAGATAGAATTCCCCCAAGATGGGAT	Amplification of sequences encoded mutated *ABI4*
4	TAP	ATGTACCCATACGATGTTCCTGAC	AAGCTTGATATCAGCGTAATCTGGA	RT-qPCR of *ABI4-eGFP*
5	18S	AAGCAAGCCTACGCTCTGGA	AGGCCAACACAATAGGATCGA	RT-qPCR of 18S rRNA
